# Photochemical Oxidative Cyclisation of Stilbenes and Stilbenoids—The Mallory-Reaction

**DOI:** 10.3390/molecules15064334

**Published:** 2010-06-14

**Authors:** Kåre B. Jørgensen

**Keywords:** Mallory-reaction, oxidative photocyclization, stilbene, iodine, photochemistry

## Abstract

After Mallory described in 1964 the use of iodine as catalyst for the photochemical cyclisation of stilbenes, this reaction has proven its effectiveness in the synthesis of phenanthrenes, other PAHs and phenacenes with a surprisingly large selection of substituents. The “early age” of the reaction was reviewed by Mallory in 1984in a huge chapter in the *Organic Reactions* series, but the development has continued. Alternative conditions accommodate more sensitive substituents, and isomers can be favoured by sacrificial substituents. Herein the further developments and applications of this reaction after 1984 are discussed and summarized.

## 1. Introduction

The oxidative photocyclizations of stilbenes was discovered earlier during studies of the photochemical isomerization of stilbenes [1,2], but the reaction did not become feasible as a synthetic tool until Mallory discovered in 1964 that iodine could catalyze the reaction [3,4]. That allowed for more concentrated solutions and fewer side reactions. The reaction was thoroughly reviewed by Mallory in a large chapter in Organic Reactions in 1984 [5]. Other reviews [6,7,8,9,10] discuss various aspects and applications of the reaction. This review will focus on the reaction as a useful tool in synthesis, covering developments reported since 1984.

## 2. Oxidative Photocyclization

**Scheme 1:**
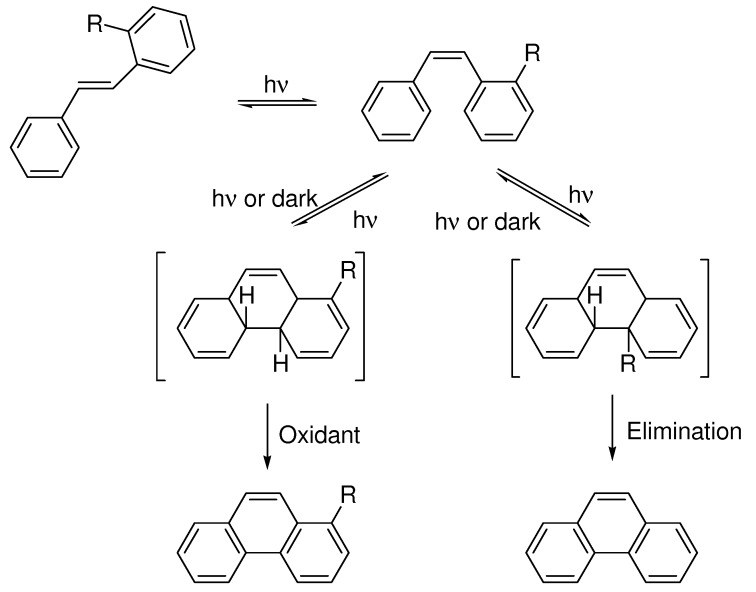
Reaction pathways for the photocyclization of stilbenes.

The reaction pathway of what should be called the Mallory-reaction is pictured in Scheme 1. Photochemical isomerization of the double bond in stilbene has been extensively studied [8,11]. From a synthetic point of view the *cis /trans*-isomerization occurs rapidly under the reaction conditions in such a way that different compositions of *cis*- and *trans*-stilbenes still give the same products. Thus, the stilbenes can be used as isomeric mixtures in the photocyclization, although only the *cis*-isomer is capable of the further cyclization. The formed dihydrophenanthrene is unstable and will, unless trapped, relax back to the stilbene. There are also examples of hydrogen-shifts at this stage under non-oxidative conditions [5]. The dihydrophenanthrene can be trapped by oxidation to form a phenanthrene, or by elimination given a suitable substituent in the *ortho*-position on one of the aromatics.

In more concentrated solutions the stilbenes can form dimers in a [2+2] cycloaddition as well [5,8,12]. Mallory discovered that this oxidative trapping occurs much faster when traces of iodine were used together with O_2_ [3], but increased concentrations of iodine did not affect the reaction rate. It has been proposed [5] that iodine is photochemically cleaved into radicals that react in a chain reaction: 


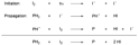


**Scheme 2:**
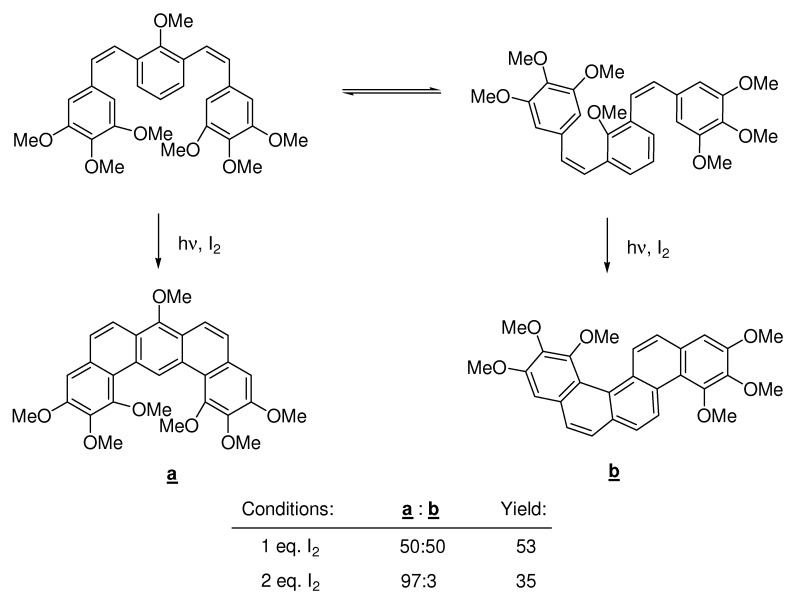
More iodine can prevent eliminative cyclization [13].

The hydrogen iodide is then oxidized back to iodine by oxygen. The reaction was compatible with fluoro, chloro, bromo, methoxy, methyl, trifluoromethyl, phenyl and carboxyl, but not nitro, acetyl or dimethylamino substituents. Iodo substituents were lost during the reaction. Concentrations were usually 0.01 mole/liter of stilbene [4]. Higher concentrations lead to more [2+2] cycloaddition between two stilbenes. The concentration of iodine can influence both product yields and product selectivity. A full equivalent of iodine per cyclization can prevent elimination of methanol [13] (Scheme 2).

On the other hand, the increased iodine concentration leads to formation of more hydrogen iodide that can saturate the starting stilbene and also contribute to other side reactions [14]. Other oxidants besides iodine have also been used, as reviewed by Laarhoven [10], but do not appear to have been preferred for carrying out syntheses. Representative examples of the Mallory-reaction published after 1984 are shown in Appendix 1.

## 3. Katz’s Conditions

Although increased amounts of iodine result in better yields in some systems, the increased concentration of hydrogen iodide causes side-reactions that limit the yields. Katz’s group officially introduced new conditions in 1991 [14] to solve this problem by scavenging the formed hydrogen iodide with methyloxirane to prevent the side-reactions (they published the first reactions with methyloxirane as a scavenger in 1986 [15,16]). As a consequence the iodide could not be reoxidized by oxygen, so one equivalent of iodine was needed and the reaction could then be performed under an inert atmosphere preventing side reactions with oxygen. The combination of hydrogen iodide and light can reduce the double bond in stilbene to a saturated bond [14]. It was observed from the beginning [3,4] that catalytic amounts of iodine gave purer products and higher yields for many systems. It is not oxygen itself that is the destructive agent, but rather substances formed from oxygen during the photocyclization [14]. Table 1 compares the yields between the use of catalytic amounts of iodine and the Katz-conditions.

**Table 1:** Comparison between catalytic iodine/oxygen and Katz’s conditions. Most examples are from ref [14].

Starting material	Product	Cat. I_2_	Katz’s conditions
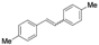	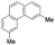	51%	95%
		(8 h)	(8 h)
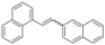	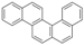	61%	100%
		(4 h)	(1 h)
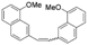	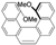	<8%	61%
		(3.5 h)	(13 h)
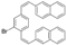	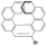	66%	87%
		(1.2 h)	(1.2 h)
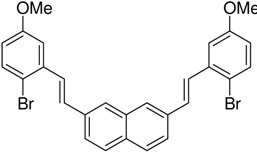	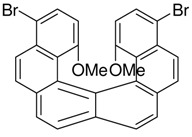	<4%	71%
		(4.5 h)	(4.5 h)
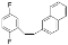	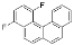	64%	71%
		Ref [17]	Ref [18]

**Scheme 3:**
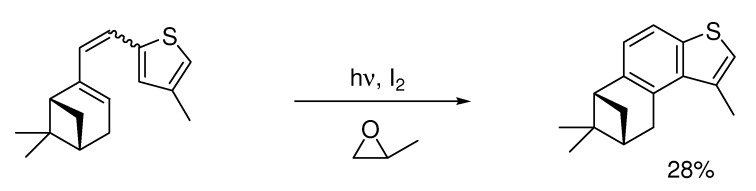
Photochemical cyclization of a less reactive molecule.

Less reactive molecules that remain unreactive in other photochemical conditions sometimes react under Katz-conditions [19], as shown in Scheme 3, below.

**Scheme 4:**
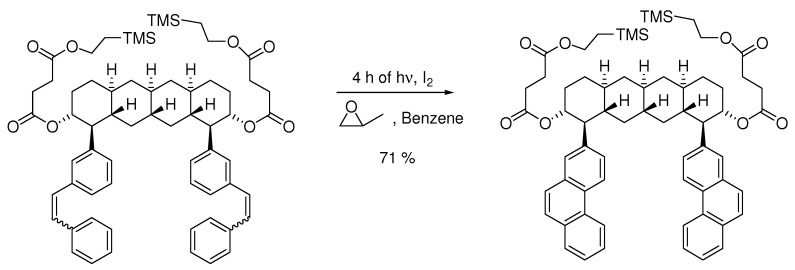
Example of highly functionalized molecule that is compatible with the Mallory condition under Katz’s conditions [26].

**Scheme 5:**
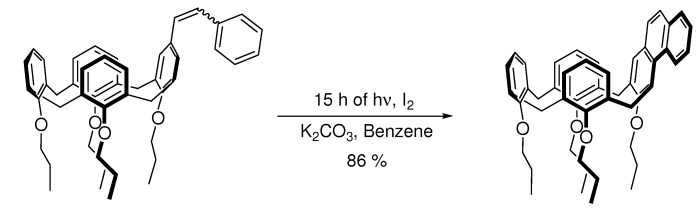
Potassium carbonate as HI-scavenger [25].

The conditions are compatible with a wide range of functionality, as illustrated in Scheme 4. The high concentration of iodine allows for a higher concentration of starting materials in the reaction without formation of dimers as a side-reaction. This is illustrated in some patents [20,21,22] describing a photocyclization with 5 g starting material per liter of solvent. Also in our experience this is about the concentration limit to avoid significant side reactions under Katz-conditions [23]. Reaction times depend on concentrations, but Katz’s conditions are often faster than using catalytic amounts of iodine [24]. Recently, potassium carbonate has also been introduced as a HI-scavenger to prevent ring opening of the alkyl chains [25] (Scheme 5). Further examples of reactions with Katz-conditions are given in Appendix II.

## 4. Elimination Photocyclizations

**Scheme 6:**
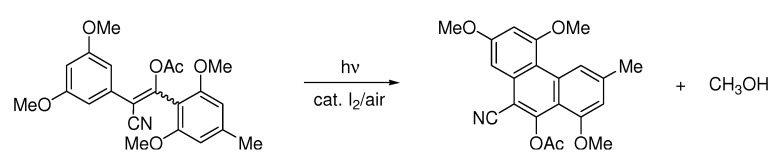
Elimination of either *orto*-methoxy-group gave the same product.

The original I_2_/O_2_-conditions sometimes give significant amounts of byproducts from elimination of *o*-methoxy-groups on the stilbenes [27]. Finnie [28] avoided the problem of elimination of methanol by putting methoxy-groups at both *ortho*-positions (Scheme 6).

However, with less symmetrical starting materials this is not a viable approach. Katz-conditions will usually reduce the problem of this kind of elimination.

**Scheme 7:**
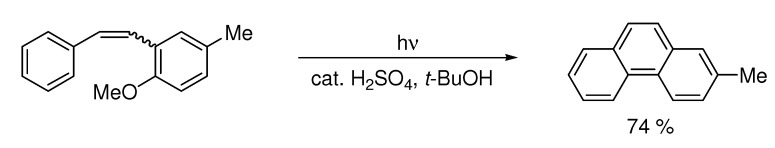
Eliminative photocyclization used to avoid the selectivity-problem with substituents in meta-position on the stilbene [29].

Mallory [29] tested acidic conditions to promote elimination of methanol to control the selectivity. The reactions needed much longer reaction times. Some selectivity towards elimination was achieved with catalytic amounts of sulfuric acid, at the cost of lower yields than with oxidative conditions on the same stilbenes. The reactions were not inverted in all cases, but a good selective synthesis of 2-methyl-phenanthrene and 4-methylphenanthrene was obtained (Scheme 7). Oxidative cyclization of meta-methylstilbene gives a 1:1 mixture of these regioisomers that are difficult to separate. However, attempts to control the cyclization into the unfavored benzo[a]anthracene failed.

It is also possible to put a good leaving group like tosyl at the bridge-double bond to promote cyclization under basic conditions [30]. Although this gave very good yields, it does not help to control the selectivity of the cyclization.

**Table 2:** Comparison of product formation between oxidative and basic elimination conditions [31].

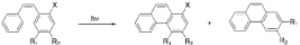
**X**	**R_1_**	**R_2_**	**Conditions**	**a**	**b**	**Product ratio**
Cl	CH_3_	H	Oxidative	95	0	>20
			Basic	8	31	4.0
Br	CH_3_	H	Oxidative	65	0	>20
			Basic	16	20	1.3
Br	OCH_3_	H	Oxidative	71	7	10
			Basic	10	41	4.1
Br	OCH_2_O		Oxidative	63	12	5.3
			Basic	0	57	>20

Dehydrobromination under basic conditions has been extensively studied, but it also has its limitations. Olsen [31] did a comparison between oxidative photocyclizations (2 equivalents of iodine) and elimination photocyclizations with NaOMe in methanol. Some of the results are summarized in Table 2. The yields of oxidative conditions were consistently higher, but some debrominations were observed as secondary reactions occurring after the cyclization. The basic conditions did change the selectivity, but to a lesser degree than desired. As can be seen in the last example, basic conditions can give very good selectivity, but only when the system is already inclined to react that way. Some more examples of eliminative photocyclizations are summarized in Appendix 3. However, this approach has generally been of limited use.

## 5. Reactivity Parameters

**Scheme 8:**
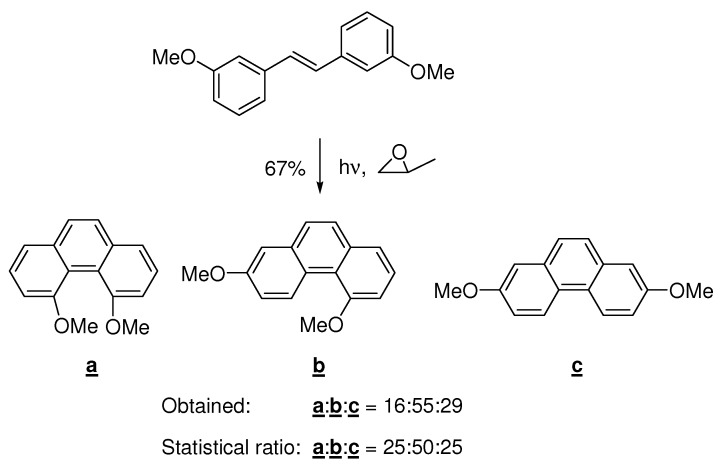
Oxidative photocyclization with two *meta*-substituents. The product composition deviates only a bit from a statistical distribution and towards less steric hindrance [32].

The Mallory-reaction is somewhat sensitive to steric effects of the substituents, as shown in Scheme 8, although the product distribution does not deviate much from a statistical distribution.

**Scheme 9:**
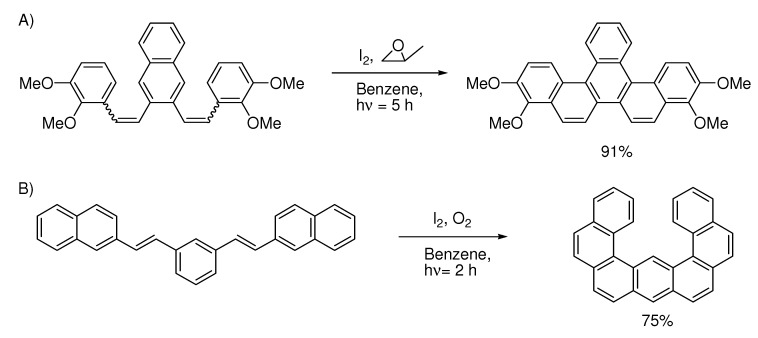
Oxidative photocyclization often gives one main regioisomer. A) Ref. [33], B) Ref. [34].

In contrast, the aromatic ring-structure regioselectivity of the Mallory-reaction is very strong. Usually only one ring-structure is formed, even when the formation of several structures look plausible. The reaction favors ring-structures that are curled towards helicene structures as the two examples in Scheme 9 show.

Laarhoven [6,10] has evaluated reactivity parameters like free valence numbers [35] (∑F^*^_rs_) and localization energies (∑L^*^_rs_) for a large number of examples. He found a good correlation between these two parameters, but found free valence numbers more convenient as only one calculation is needed to evaluate all cyclization modes of a particular compound. ∑F^*^_rs_ is the sum of the free valence numbers of atoms *r* and *s* involved in the cyclization in the exited state (F*_r_*= √3-∑P in which P is the bond order).

Three rules [6] for cyclization were determined:

(i) Photocyclizations do not occur when ∑F^*^_rs_<1.0.
(ii) When two or more cyclizations are possible in a particular compound, only one product arises if ∆(∑F^*^_rs_) > 0.1; more products are formed if the differences are smaller.
(iii) The second rule holds when only planar or non-planar products (penta- or higher helicenes) can arise. When planar as well as non-planar products can be formed, the planar aromatic in general is the main product, provided that for its formation ∑F^*^_rs_> 1.0

**Scheme 10:**
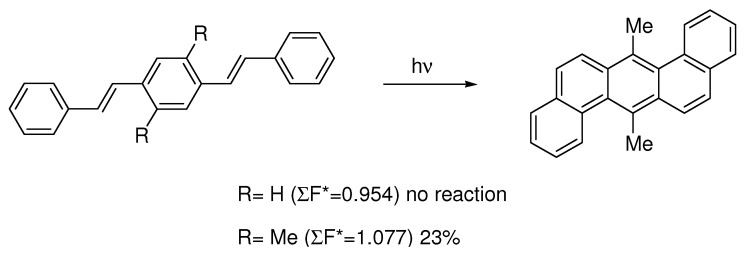
Calculation of ∑F^*^_rs_ for the reaction indicates that methyl-substituted distyrylbenzene can undergo photocyclization, but not the unsubstituted compound. Experiments are in accordance with this [6,36].

Photocyclization of 1,4-distyrylbenzene [6] is a good example (Scheme 10). Another example applies to 1,3-distyrylbenzene [8]. The best discussion with several reaction examples with calculated reactivity parameters is given in a review by Laarhoven [10]. These rules should be a useful planning tool for synthesis, but no examples have been found where these rules have actually been applied in the such planning. One reason might be that the theory and calculations of these reaction parameters are not very accessible for the typical synthesis chemist with a limited background in theoretical chemistry.

## 6. Controlling Product Formation with Blocking Groups

**Scheme 11:**
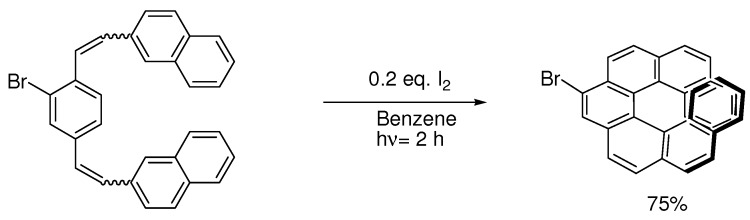
Br is used as a blocking group.

**Scheme 12:**
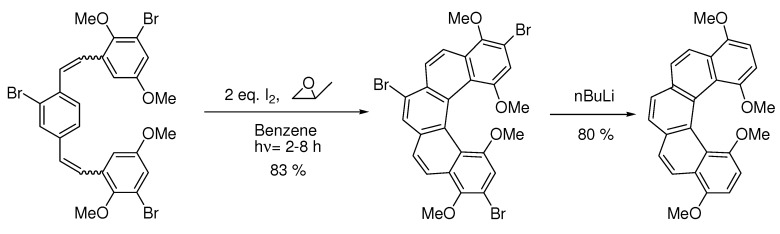
Br also protects neighbouringmethoxy-groups from elimination-cyclization.

**Scheme 13:**
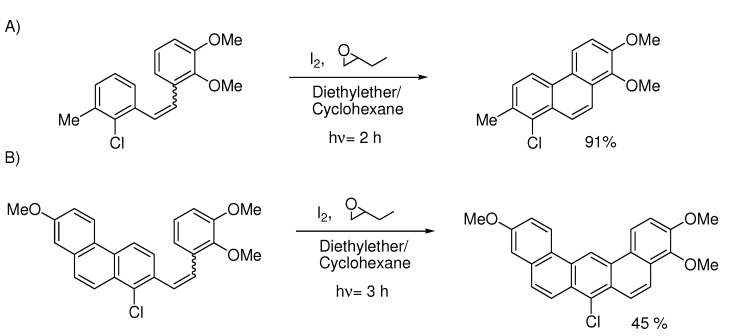
Examples from ref. [43]. In A) the reaction follows the natural cyclization path but the chloro-group prevents the 50:50 product mixture from *meta*-methyl. In B) the chloro-groups blocks the preferred cyclization path and forces the product formation.

Helicenes are borderline molecules in Laarhoven’s cyclization rules. When they become larger than five benzene rings they become non-planar, and thus no longer favored products. Formation of planar S-shaped molecules becomes the main side reaction or even the main reaction. This led Katz’s group to develop the bromo-group as a directing substituent [15,16,37]. The bromo-group also blocks its neighbor position in the cyclization (Scheme 11):

Without the bromo-group the reaction gives 1:1 [7]helicene and the S-shaped benzo[a]naphto[1,2-k]tetraphene [15]. Without blocking groups the yield of [6]helicenes also becomes low [38]. The bromo-group can even protect neighbouring methoxy-groups from elimination-cyclization. Without the bromo-groups in the example below a mixture of the desired product and products resulting from elimination reactions occurred [37] (Scheme 12):

It has proven more difficult to use blockers to change the regioselectivity into anthracene-like moieties. Amin [39] experienced low yields and further oxidation of the bromo-group into quinones while trying to force the reaction away from benzo[c]phenanthrene and towards benzo[a]anthracene. Harvey tested different blocking groups and concluded that the chloro-group works better [40]. There is still a price to pay for working against the natural pathway as illustrated in Scheme 13. In PAH-synthesis the Mallory-reaction encounters competition from other methods for several ring-systems [41,42].

The overlapping helicenes are chiral, and have very large specific rotation ([α]^D^_25_ = 3640° for [6]helicene [44]). This allowed for a study of the small enantioselectivities induced by chiral solvents during the Mallory-reaction [44].

**Scheme 14:**
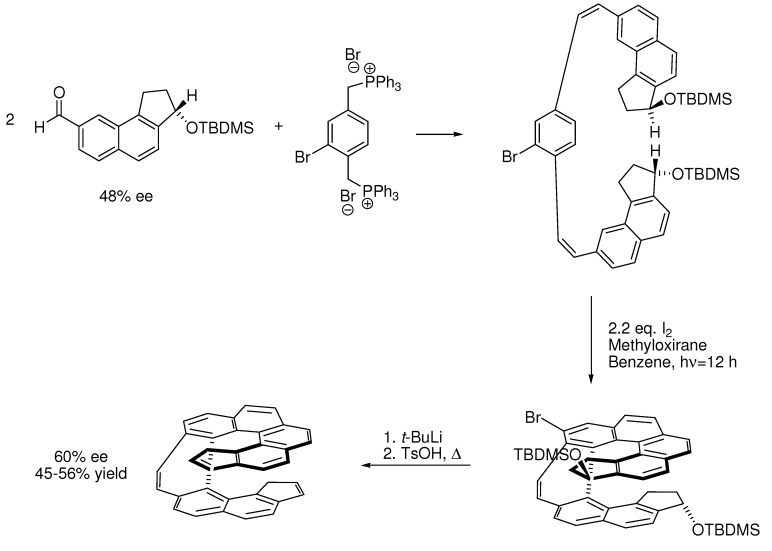
Synthesis of a chiralhelicene-system obtained with a double Mallory-reaction with Br as a blocking group. The two chiral groups get placed on the outside of the helicene to avoid unnecessary bending of the aromatic system [16].

Inflexible chiral groups on the substrate for a double Mallory-reaction gave a [7]helicene with better enantiomeric excess than the starting material (Scheme 14) [16]. The use of more flexible chiral auxiliaries like menthol on a carboxylic acid substituent gave lower diastereoselectivities in the formation of [5]- and [6]helicenes [45].

**Scheme 15:**
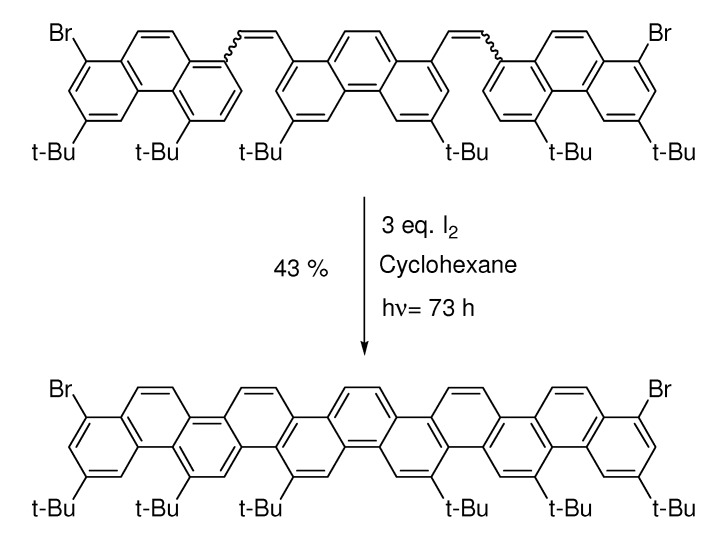
Photochemical synthesis of phenacenes.

The reaction has lately also made its way into material science [46,47]. It is appropriate to end this review with a series of papers by Mallory [48,49,50], the latest 37 years after the publication of the use of iodine as a catalyst [3]. Here [48] steric hindrance allows formation of carbon-ribbons (phenacenes) (Scheme 15):

## Appendix 1

**Appendix 1:** Oxidative photocyclization, original conditions.

Staring material	Conditions		Products	Reference	
	0.3 eq. I_2_, Cyclohexane, hν = ?			[51]	
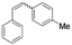
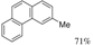
	0.5 eq. I_2_, Cyclohexane, hν = 24 h (42 mmol/L)			[52]	
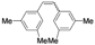
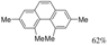
	Cat. I_2_, Ethanol, hν = 8 h			[53]	
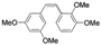
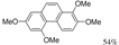
	0.67 eq. I_2_, Cyclohexane, hν = 47 h			[54]	
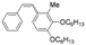
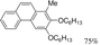
	0.3 eq. I_2_, Cyclohexane, hν = ?			[51]	
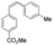
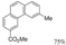
	Cat. I_2_, Toluene, hν = 24 h			[55]	
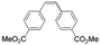
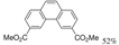
	0.5 eq. I_2_, Toluene, hν = 3 days			[56]	
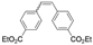
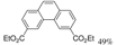
	Cat. I_2_, Methanol, hν = 30 h			[57]	
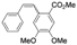
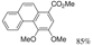
	1 eq. I_2_, Diethylether/ DCM, hν = ?			[58]	
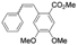
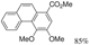
	Cat. I_2_, Cyclohexane, hν = 7 h			[59]	
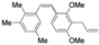
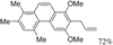
	Cat. I_2_, Cyclohexane, hν = 2 h			[59]	
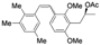
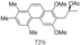
	Cat. I_2_, Diethylether, hν = 3 h			[60]	
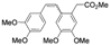
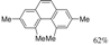
	Cat. I_2_, Diethylether, hν = 3 h			[60]	
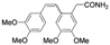
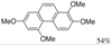
	Cat. I_2_, Diethylether/ DCM, hν = 2 h			[57]	
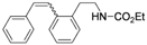
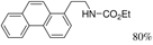
	Cat. I_2_, Diethylether/ DCM, hν = 5 h			[61]	
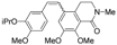
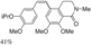
	Cat. I_2_, Diethylether, hν = 2 h			[62]	
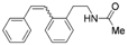
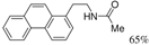
	Cat. I_2_, Cyclohexane, hν = 3 h			[63]	
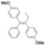
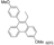
	Cat. I_2_, DCM/ Cyclohexane, hν = 1 h			[64]	
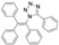
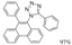
	0.25 eq. I_2_, Biacetyl, Toluene, hν = 40 min.			[65]	
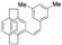
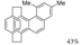
	Cat. I_2_, Cyclohexane, hν = ?			[66]	
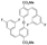
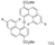
	Cat. I_2_, Cyclohexane, hν = ?			[67]	
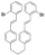
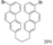
	Cat. I_2_, Toluene, hν = 12 h			[68]	
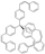
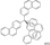
The free acid did not react.	Cat. I_2_, Benzene, hν = 24 h			[69]	
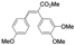
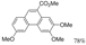
	Cat. I_2_, Benzene, hν = 2 days			[69]	
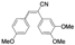
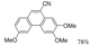
	Cat. I_2_, Methanol, hν = 21 h			[70]	
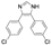
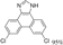
		2 eq. I_2_, Benzene, hν = 8 h		[71]	
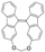
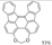
		Cat. I_2_, Benzene, hν = 24 h		[72]	
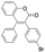
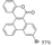
		0.5 eq. I_2_, Benzene, hν = 15 h		[73]	
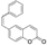
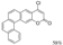
		0.5 eq. I_2_, Benzene, hν = 36 h		[73]	
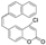
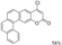
		Cat. I_2_, Benzene, hν = 4 h		[74]	
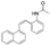
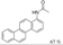
		Cat. I_2_, Benzene, hν = 12 h		[47]	
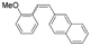
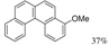
		Cat. I_2_, Cyclohexane, hν = ?		[75]	
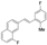
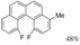
		Cat. I_2_, Cyclohexane, hν = ?		[75]	
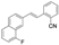
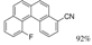
		Cat. I_2_, Cyclohexane, hν = 40 h		[76]	
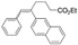
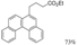
		Cat. I_2_, Benzene, hν = 7 days		[77]	
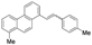
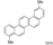
		Cat. I_2_, Acetone, hν = 16 h		[78]	
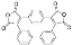
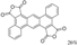
		1 eq.I_2_, Toluene/Hexanes, hν = 60 h		[48]	
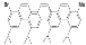
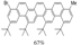

**Appendix 2:** Oxidative photocyclization, Katz’s conditions.

	Staring material		Conditions		Products		Reference	
			I_2_, Methyloxirane, Toluene, hν = 1.5 h				[38]	
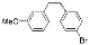
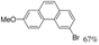
			I_2_, Methyloxirane, Toluene, hν= 4 h				[32]	
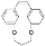
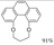
			I_2_, Methyloxirane, Benzene, hν = 3 h				[79]	
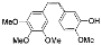
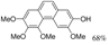
			I_2_, Methyloxirane, Cyclohexane, hν = 4 h				[24]	
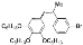
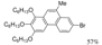
			I_2_, Methyloxirane, Cyclohexane, hν = 12 h				[80]	
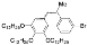
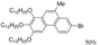
			I_2_, Methyloxirane, Toluene, hν = ?				[81]	
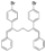
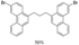
			I_2_, Methyloxirane, Toluene, hν = ?				[82]	
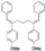
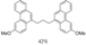
			I_2_, Methyloxirane, Benzene, hν = 40 h				[83]	
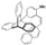
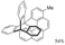
			I_2_, Methyloxirane, Light petroleum, hν = 2 h				[84]	
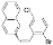
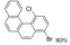
			I_2_, Epoxybutane, Toluene, hν = 1.5 h				[23]	
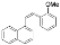
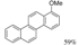
			I_2_, Epoxybutane, Benzene, hν = 2 h				[85]	
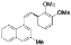
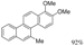
			I_2_, Methyloxirane, Cyclohexane, hν = 50 h				[86]	
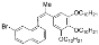
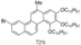
			I_2_, Epoxybutane, Benzene, hν = 8 h				[87]	
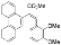
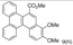
			I_2_, Methyloxirane, Benzene, hν = 5 h				[88]	
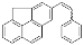
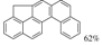
			I_2_, Methyloxirane, Benzene, hν = 6 h				[89]	
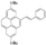
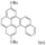
			I_2_, Epoxybutane, Diethylether/ Cyclohexane, hν = 8 h				[90]	
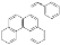
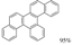
			I_2_, Methyloxirane, Benzene, hν = 12 h				[46]	
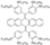
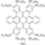

**Appendix 3:** Elimination photocyclizations.

Staring material	Conditions	Products	Reference	
	Cat. H_2_SO_4_, t-BuOH/Benzene, hν = 175 h		[29]	
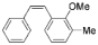
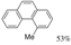
	Cat. H_2_SO_4_, t-BuOH/Benzene, hν = 26 h		[29]	
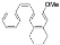
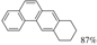
	5 eq. DBU, THF, hν = 11 h		[30]	
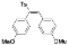
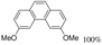
	5 eq. DBU, THF, hν = 6.5 h		[30]	
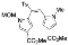
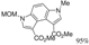
	t-BuOK, t-BuOH/Toluene, hν = 6 h		[31]	
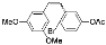
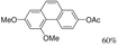
	t-BuOK, t-BuOH/Toluene, hν = 8 h		[91]	
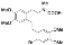
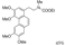
	t-BuOK, t-BuOH/Toluene, hν = 10 h		[92]	
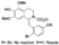
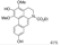
	t-BuOK, t-BuOH/Toluene, hν = 15 min. (?)		[93]	
